# Long-Term Outcomes After Open Repair for Ruptured Abdominal Aortic Aneurysm

**DOI:** 10.1007/s00268-020-05457-7

**Published:** 2020-03-09

**Authors:** Andreas Reite, Kjetil Søreide, Jan Terje Kvaløy, Morten Vetrhus

**Affiliations:** 1 Department of Surgery, Vascular Surgery Unit, Stavanger University Hospital, PO Box 8100, 4068 Stavanger, Norway; 2 Department of Gastrointestinal Surgery, Stavanger University Hospital, Stavanger, Norway; 3 Department of Clinical Medicine, University of Bergen, Bergen, Norway; 4 Research Department, Stavanger University Hospital, Stavanger, Norway; 5 Department of Mathematics and Physics, University of Stavanger, Stavanger, Norway; 6 Department of Clinical Science, University of Bergen, Bergen, Norway

## Abstract

**Background:**

Early mortality in ruptured abdominal aneurysm (rAAA) is high, but data on long-term outcome are scarce. The aim of this study was to investigate the long-term outcome in survivors after open surgery for rAAA in well-defined population.

**Methods:**

This is a population-based, observational long-term follow-up (beyond 30-day mortality) study of patients surgically treated for rAAA from 2000 through 2014. Long-term survival was analysed using Kaplan–Meier estimates and compared to the general population by analyses of relative survival.

**Results:**

Out of 178 patients operated for rAAA, 95 patients (55%) either died in the perioperative period, were referred from other hospitals or were lost to follow-up (two patients). Altogether 83 patients were eligible for long-term outcomes: 72 men and 11 women. Estimated median crude survival time was 6.5 years [95% confidence interval (CI) 4.8–8.2]. Men had a median survival of 7.3 years (95% CI 5.1–9.4) versus 5.4 years in females (95% CI 3.5–7.3) (*P *= 0.082). Reinterventions during follow-up occurred in 31 (37%). Relative survival demonstrated a slightly higher risk of death in the rAAA population compared to the general age- and gender-matched population. Age, but not comorbidities, had a significant influence on long-term survival.

**Conclusion:**

For survivors beyond 30 days after surgery for rAAA, long-term survival compares well to that of an age- and sex-matched population. A high frequency of cardiovascular comorbidities did not seem to affect long-term survival.

## Introduction

Surgery for ruptured abdominal aortic aneurysms (rAAA) is still challenging with perioperative mortality rates ranging from 40 to 60% in most studies [1, 2]. Reports suggest a wide variation in perioperative mortality [2, 3]. The incidence of rAAA and the perioperative mortality has declined over the last two decades, and lower mortality may be due to the implementation of endovascular repair and improved pre- and in-hospital care [2, 4, 5]. Previous studies have mainly focused on the perioperative period, and less attention has been paid to the long-term outcome in patients who do survive the first 30 days. The latest Cochrane review [6] stated that long-term data on survival and late complications are scarce after both endovascular and open treatments of rAAA. Factors that may affect survival past the perioperative period in patients operated for rAAA have been described in a few previous reports, and the results are not consistent [7, 8]. Few population-based studies on long-term outcome with an adequate follow-up time are available [9]; the majority of these studies are from a pre-selected population, i.e. not population-based, and thus, the generalizability of the findings is questionable.

The aim of this study was to present data on long-term survival, including relative survival and factors related to long-term survival and excess mortality in a well-defined Norwegian population.

## Methods

The study was approved by the Regional Committee for Medical and Health Research Ethics North (REK Nord 2011/918).

### Study population

Stavanger University Hospital (SUH) has a primary catchment population of 360,000 and is the only hospital serving the population of South Rogaland County. SUH also receives tertiary referrals from other hospitals, covering a total population of about 500,000 inhabitants. The epidemiology and perioperative mortality for rAAA in this region have been reported earlier [1, 10].

### Study design and inclusion criteria

The study is a single-centre population-based retrospective observational study, including all patients from SUHs primary catchment area operated for rAAA during the period from January 2000 to December 2014. The study was confined to juxta-renal and infra-renal primary rAAA only. To avoid referral or survival bias from transferred patients, only patients from the primary catchment area were included.

Survival and mortality data were obtained from the prospectively maintained electronic patient record database at the hospital. The study has, to the best of our abilities, been conducted according to the STROBE guidelines for observational studies [11].

### Definitions

rAAA was confirmed by extravasation of blood on computed tomography (CT) and/or a periaortic haematoma on operation. Secondary aneurysms (anastomotic aneurysms after the previous repair of aneurysm or atherosclerosis) and isolated iliac aneurysms without any aortic component were excluded.

Comorbidity was defined as evidence in past medical history of organ or system diseases. Heart disease included any heart condition including congestive heart failure, rhythm disturbances and previous ischaemic heart disease.

The patients’ physical status was classified according to the American Society of Anaesthesiologists (ASA) Physical Status Classification [12] into five classes where class 1 is a normally healthy patient and class 5 is a moribund patient who is not expected to survive for 24 h with or without operation.

Complications after surgery were classified as minor or major, major defined as complications requiring reintervention under general anaesthesia or the presence of multiorgan failure.

The Oxford Vascular study [13] showed that two-thirds of acute abdominal aortic aneurysm (AAA) events occur at age ≥75 years; we used this age as a cut-off to dichotomize patients into ‘older’ (≥75 years) or ‘younger’ (<75 years).

### Survival data

Survival and mortality data were obtained from the electronic patient record database where vital status is regularly updated from the Norwegian national population data registry. Patients alive as per 6 October 2018 were censored.

### Statistical analyses

All analyses were performed with the Statistical Package for Social Sciences (v 23; IBM, Armonk, NY, USA) and R 3.4.3 [14] The R package ‘relsurv’ version 2.0-9 was used for the relative survival calculations [15].

Mann–Whitney test was used to test for differences between continuous variables and Pearson’s Chi-squared test for categorical values.

Relative survival analysis was calculated as the probability of patients operated for rAAA (excluding rAAA with 30-day mortality) surviving to a given time post-operatively divided by the probability that persons in the general population would survive to that time, given the same age, gender and year of birth distributions. Population survival was calculated with Norwegian population lifetime tables from Statistics Norway [16].

For crude survival calculations, Kaplan–Meier estimates were used and investigated by the log-rank test. For impact of covariates, first univariable Cox regression was performed. Then covariates with *P *< 0.2 were included in a multivariable Cox regression analysis.

All statistical tests were two-sided, and the level of significance was set at *P *< 0.050.

## Results

**Fig. 1:**
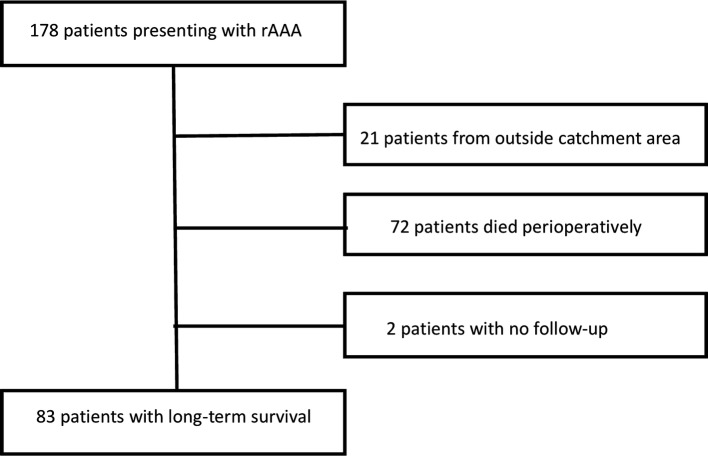
Patient population with selection criteria

During the study period, 178 patients had an operation for rAAA (Fig. 1). All but one patient (endovascular repair) had an open operation.

Patients who died perioperatively (within 30 days) were excluded (*N* = 72) from the analyses. Patients referred from other institutions with rAAA (*N* = 21) were excluded for a correct epidemiological assessment. Finally, two patients had no follow-up data as they had left Norway. Thus, 83 patients were included in the study: 72 (87%) males and 11 (13%) females (Table 1). Altogether, 58 of 83 patients (70%) died during follow-up.

**Table 1:** Patient characteristics stratified by gender

Variables	Male (*N* = 72)	Female (*N* = 11)	*P*
Age	72.8 (9.9)	84.1 (8.9)	0.021
Aneurysm size (mm)	76.0 (18.1)^a^	72 (18.9)	0.615
Survival (year)	5.8 (4.5)	5.4 (3.1)	0.184
Intensive care (days)	5 (15.6)	2 (12.6)	0.137
Ventilator (days)	1 (12.8)	0 (5.6)	0.186
Bleeding (mL)	2400 (2602)	2400 (3132)	0.893
Operation time (min)	158 (63.6)	143 (59.0)	0.379
Preoperative pulse rate	67 (16.4)	60 (15.2)	0.294
Preoperative systolic blood pressure (mm Hg)	70 (15.7)	80 (11.4)	0.205
Blood transfusion (mL)	1750 (1375)	1750 (839)	0.877
Heart disease	23 (32%)	7 (64%)	0.042
Coronary disease	18 (25%)	6 (55%)	0.044
Pulmonary disease	7 (10%)	0	0.280
Cerebrovascular disease	6(8%)	3 (27%)	0.060
Renal failure	3 (4%)	0	0.490
Diabetes Mellitus	6 (8%)	1 (9%)	0.933
Beta-blocker	10 (14%)	2 (18%)	0.706
Statins	24 (33%)	3 (27%)	0.689
ASA	4 (2–5)^b^	4 (3–4)	0.590

Data presented as counts (%) or median (±SD). All values are median with range or percentage in parentheses. Missing data: a: six cases, b: three cases

### Patient characteristics

Patient characteristics are given for females and men in Table 1. A substantial number of the patients had known heart disease: 31% (*N* = 13) of the patients <75 years and 45% (*N* = 17) of the elderly patients. Increasing age was associated with mortality (Table 2). No significant difference in comorbidity or rate of new interventions was found in a comparison between the different age groups.

**Table 2:** Risk factor analysis for death in rAAA (30-day mortality excluded)

Variables	Hazard ratio (95% CI)	*P*
Age	1.1 (1.0–1.1)	0.002
Sex	2.0 (1.0–4.1)	0.066
Coronary heart disease	0.9 (0.5–1.5)	0.604
Renal failure	2.7 (0.4–19.5)	0.327
Pulmonary disease	0.4 (0.2–1.1)	0.093
Cerebrovascular disease	0.5 (0.2–1.2)	0.121
Diabetes mellitus	0.9 (0.3–2.4)	0.866
Transfusion (SAG) >1200 mL	0,9 (0.5–1.5)	0.608
Statins	0.9 (0.5–1.6)	0.747
Beta-blocker	2.7 (0.8–8.5)	0.101

### Complications, reinterventions and mortality

Early (<30 days) and late complications leading to surgical intervention are listed in Table 3: 24 early complications were registered in 18 patients (22%) as four patients needed intervention for more than one complication.

**Table 3:** Early and late complications requiring surgical intervention, counts (%)

Complications requiring intervention	*N* = 83	Mean time to intervention
Early (<30 days)		
Major amputation	5 (6%)	11 days
Colectomy, partial or total	5 (6%)	7 days
Open abdomen	3 (4%)	1 day
Haemorrhage	5 (6%)	8 days
Wound dehiscence	3 (4%)	3 days
Revascularization lower limb	3 (4%)	2 days
Late (>30 days)		
Secondary rupture	1 (1%)	8 years
Ventral hernia	2 (2%)	1.5 years
Graft infection	2 (2%)	0.5 year

Late complications were registered in five patients (6%). One patient had a secondary rupture in the distal anastomosis that was successfully repaired, and two patients (2%) were diagnosed with graft infection that presented with a psoas abscess adjacent to the aortic graft; one patient died from septicaemia, while the other was successfully treated with antibiotics.

The cumulative number of reinterventions ranged from 1 to 4 per patient. The total number of reinterventions per patient had a significant impact on long-term survival (log rank, *P *= 0.009).

Most patients experienced a temporary decrease in renal function with a median creatinine before surgery of 113 μmol/L (range 53–500) and 145 μmol/L (range 56–677) after surgery. Eleven (13%) patients had more than a threefold raise in creatinine after surgery, equivalent to acute renal failure. Four (5%) patients developed persistent renal failure requiring permanent renal replacement therapy. Altogether 51 (61%) patients were discharged to their home, 24 (29%) to a short time stay in a nursing home and eight (10%) to another hospital. The long-term functional outcome is not known.

The cause of death was known in 38 patients who died in hospital (Table 4): 32 (84%) men with a median age of 74 years and 6 (16%) females with a median age of 79 years. Seven of the 83 patients (8%) included in the study died of aneurysm-related causes. Four (11%) patients died between 30 and 90 days after surgery from renal, cardiac and/or pulmonary complications of rAAA.

**Table 4:** Cause of death in the 58 patients who died during follow-up (>30 days)

Cause of death	*N* = 58
Cancer	13
Heart disease	6
Lung disease*	6
Cerebrovascular accident**	4
Dementia	1
Metachronous primary aneurysm	2
Post-operative complications, 30–90 days after surgery	4
Graft infection	1
Pancreatitis	1
Unknown	20

*Pneumonia or COPD-related, **stroke or intracranial haemorrhage

Cancer (34%, *N* = 13), cardiovascular (including stroke, 26%, *N* = 10) and pulmonary diseases (16%, *N* = 6) were the three major causes of death in the 38 patients with a confirmed cause of death. The cause of death was unknown for all 20 patients who died outside of hospital.

### Crude, long-term and relative survival

**Fig. 2:**
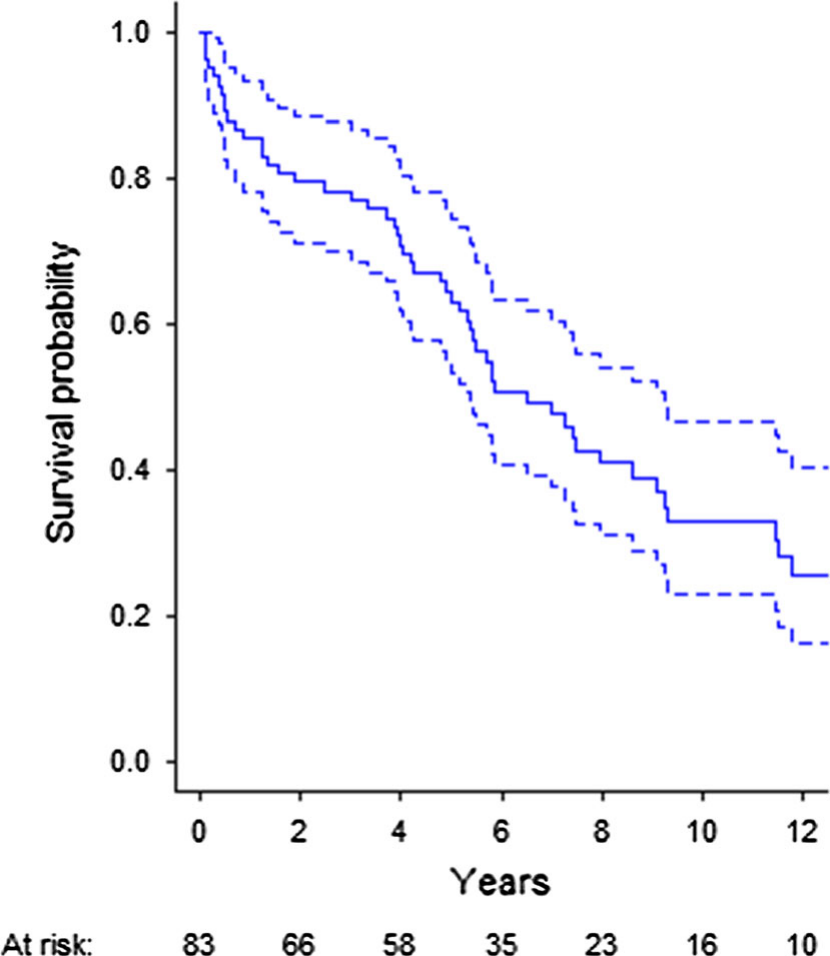
Crude survival. Legend: interval from time of operation to 12 years post-operatively. Number at risk displayed below. The solid line represents the crude survival, and the dotted lines represent 95% CI

The longest follow-up was 17.6 years, and the median follow-up was 5.7 years [95% confidence interval (CI) 4.6–7.0]. At the end of the study, 25 (30%) patients were still alive. The estimated median crude survival (excluding 30-day mortality) (Fig. 2) was 6.5 years (95% CI 4.8–8.2). For males, the median survival time was 7.3 years (95% CI 5.1–9.4), and for females, it was 5.4 (95% CI 3.5–7.3). There was a non-significant difference in long-term survival between the genders (*P *= 0.082). Hazard ratio for relevant comorbidities is displayed in Table 2.

For patients <75 years the median survival was 9.2 years (95% CI 3.6–14.9) and 5.4 years (95% CI 3.4–7.4) for those ≥75 years. The younger patients had a significantly longer survival (log rank, *P *= 0.003) (Table 2).

In patients with known heart disease, median survival was 5.9 years (95% CI 4.9–6.9), compared to 7.5 years (95% CI 3.4–11.5) in those without. The difference in survival was not significant (log rank, *P *= 0.195). Patients with lung disease had a median survival 3.9 years (95% CI 0.2–7.6) compared to individuals with no lung disease 7.0 years (95% CI 5.2–8.7), again the difference was not significant (log rank, *P *= 0.122). One patient survived 30 days after being submitted with rAAA and ongoing CPR. This patient was still alive at the end of follow-up 14 years after surgery for rAAA.

**Fig. 3:**
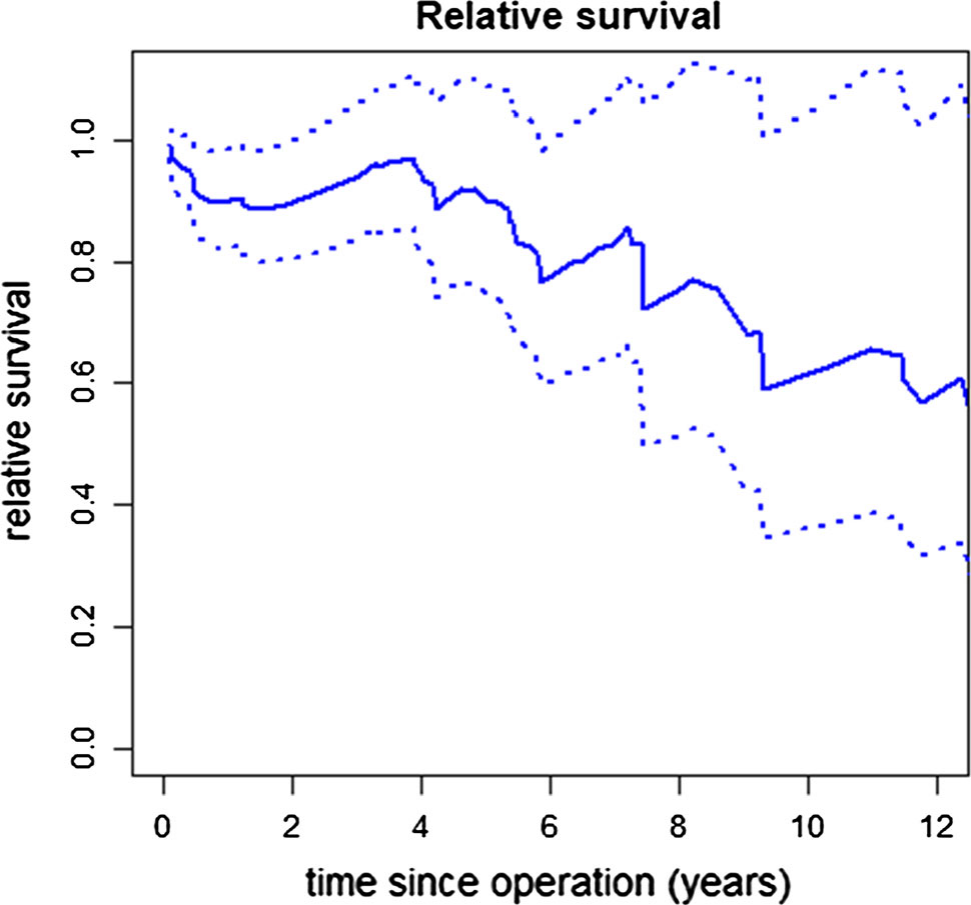
Relative survival. Legend: interval from time of operation to 12 years post-operatively. The solid line represents the relative survival, and the dotted lines represent 95% CI

The relative survival demonstrated only a slightly higher risk of death in the operated population the first 5 years after surgery compared to an age- and sex-matched general population. As follow-up time approached 12 years, the estimated relative survival was lower in patients operated on for rAAA compared to the general population, but at the extreme of follow-up few patients were at risk and the findings are uncertain due to wide confidence intervals. The 12-year relative survival is displayed in Fig. 3.

## Discussion

In this population-based observational study of patients undergoing open surgery for rAAA and surviving the first 30 days, actual survival was 64% after 5 years and 33% after 10 years. For patients surviving the first 30 days after surgery for rAAA, long-term survival was good and comparable to that of an age- and sex-matched population. When excluding mortality related to the perioperative period, age was the only factor that significantly affected survival in patients with rAAA. The durability of repair was good, with a low aneurysm-related mortality. No data on long-term functional outcome were available, but a majority of patients were discharged to their home.

The prevalence of AAA and the incidence of rAAA have been reported to decline over the last two decades [17]. Survival from rAAA has increased in the same time period. This may have a multifactorial cause including improvements in pre-hospital care, better protocols and operative management and improved intensive care post-operatively [18].

The results from previously published studies on long-term survival after rAAA outside of Norway are not consistent [8, 9, 19–28]. Several investigators have concluded that long-term outcome for patients treated surgically for rAAA was worse than the population at large [7, 20]. Vice versa, recent papers substantiate that long-term survival in rAAA is equal to or slightly lower than in the general population [8, 9, 21], and one study found that 10–16 months after surgery for rAAA the relative survival reached the same level as for an age- and gender-matched population [22].

The IMPROVE trial investigators have recently presented 3-year clinical outcome from the UK trial [29] with a three-year mortality after open repair of 54 versus 42% after endovascular repair. This is comparable to the crude survival after 3 years in our study representing a general, unselected cohort of all-comers to a single hospital (Fig. 2).

Age at time of presentation is a strong predictor of long-term survival [8]. Age was also found to be predictive of long-term survival in the current study. A study from the Swedish Vascular registry spanning 8 years found that in rAAA, both age and the proportion of patients with comorbidities increased over time, but interestingly no significant difference in long-term survival over time [21]. In a comparison of gender and long-time survival, we did not find any significant difference (*P *= 0.449). Most studies on long-term survival describe a significant difference in disfavour of women in survival [7, 8, 21]: higher expansion rate, likelihood of rupture at a smaller diameter and in general higher mortality [30, 31].

Surgical reintervention was needed in 31 patients (37%); the majority were confined to the first 30 days after primary surgery. The few late complications (5%) were registered after a median follow-up of 5.7 years and are comparable to the results from a meta-analysis of three randomized controlled trials (IMPROVE, AJAX and ECAR) [32] which reported a compiled late reintervention rate of 2.2% in open repair after a short follow-up time of 1 year (>30 days to 1 year). In the ECAR trial [33], haemodialysis beyond 30 days was reported to be 3.9% in the open group, similar to our findings.

In the present study, only one patient was treated for a paraanastomotic aneurysm (1%), and it seems reasonable to state that this is an underestimate as patients have no imaging scheduled later than 1 year after surgery. An earlier study reported the incidence of paraanastomotic aneurysms to be 15% after a mean follow-up of 7 years after surgery for rAAA [24].

The aneurysm-related mortality later than 30 days post-operatively was 7%, mainly due to deaths between 30 and 90 days from complications directly related to the surgery in a prolonged post-operative period. If excluding the four patients who died in the prolonged post-operative setting, very few patients died from aneurysm-related causes. Others have reported findings similar to ours; vascular- and graft-related mortality was higher in rAAA (3%) then after elective repair (1%) after a mean follow-up of 7 years [24]. How one defines aneurysm-related mortality is poorly defined in the literature [24, 34], and varying classifications may certainly have an impact on the numbers reported.

Cause of death is well documented in the randomized trials on rAAA [29, 33, 35], but rarely reported in other investigations on long-term survival in rAAA [8, 22, 25]. The IMPROVE trial [29] reported an aneurysm-related cause of death in 4.4%, cardiovascular 10.9%, pulmonary 8.2% and cancer 7.1%. A previous population-based study found ischaemic heart disease (36%) to be the primary cause of death in both elective and ruptured aneurysm repairs [19]. A German study reported that patients surviving the first 6 months died of the same causes as the age- and gender-matched population [36]. In the present study, none of the deaths registered after 6 months can be directly related to the prior surgical treatment of rAAA. The cause of death was known in about two-thirds of patients who died during follow-up. According to the Norwegian Institute of Public Health [37] cardiovascular, cancer and pulmonary diseases are the cause of death in 25, 25 and 10% of cases in Norway, respectively. Thus, deaths from cancer may have been overrepresented in the study group.

A high rate of cardiovascular comorbidities did not seem to affect survival, nor did other comorbidities. Others have found coronary heart disease [20, 24], but also lung disease and renal failure to be a significant factor in predicting poorer long-time survival after surgical treatment for rAAA [20, 28]. The impact of cardiovascular comorbidities on survival may have been biased by the high perioperative mortality in this study group (46%). Those with significant heart disease may have died in the initial perioperative period, thus representing a survival bias.

A Norwegian study from the early 1980s [34] reported that cardiovascular disease was the cause of death in 50% of all deaths after surgery for rAAA in the perioperative period and later. Of the patients who survived the 30 days post-surgery, 29% died from heart disease. This is in accordance with our findings, where cardiac disease was the cause of death in 26% (*N* = 6) of patients with a known cause of death.

The epidemiologically well-defined, unselected population and the long follow-up time of this study are a strength compared to similar studies on outcome after rAAA repair. Few population-based reports have been published with such a long follow-up time; most studies have a mean or median follow-up time of 1–4 years [7, 9].

The major limitation of this study is the limited size of the study population, especially the few women. Only one patient was treated with rEVAR as this procedure was generally not available in the institution during the study period. This may affect the generalizability of our findings.

Long-term survival after surgery for rAAA was good and comparable to the survival of an age- and sex-matched population. A majority of patients surviving rAAA were discharged directly to their home indicating a good functional level.
